# Revealing genes related teat number traits via genetic variation in Yorkshire pigs based on whole-genome sequencing

**DOI:** 10.1186/s12864-024-11109-0

**Published:** 2024-12-18

**Authors:** Jialin Wei, Jingchun Sun, Yi Pan, Minghao Cao, Yulong Wang, Tiantian Yuan, Ao Guo, Ruihua Han, Xiangdong Ding, Gongshe Yang, Taiyong Yu, Rongrong Ding

**Affiliations:** 1 Key Laboratory of Animal Genetics, Breeding and Reproduction of Shaanxi Province, Laboratory of Animal Fat Deposition & Muscle Development, College of Animal Science and Technology, Northwest A&F University, Yangling, 712100 Shaanxi China; 2 Key Laboratory of Agroecological Processes in Subtropical Region, Institute of Subtropical Agriculture, Chinese Academy of Sciences, Changsha, 410125 Hunan China; 3 Tongchuan Animal Husbandry Technology Extension Station, Tongchuan, 727000 Shaanxi China; 4 Key Laboratory of Animal Genetics and Breeding of Ministry of Agriculture and Rural Affairs, National Engineering Laboratory of Animal Breeding, College of Animal Science and Technology, China Agricultural University, Beijing, 100193 China

**Keywords:** GWAS, Teat number, Whole genome sequencing, Structural variation

## Abstract

**Background:**

Teat number is one of the most important indicators to evaluate the lactation performance of sows, and increasing the teat number has become an important method to improve the economic efficiency of farms. Therefore, it is particularly important to deeply analyze the genetic mechanism of teat number traits in pigs. In this study, we detected Single Nucleotide Ploymorphism (SNP), Insertion-Deletion (InDel) and Structural variant (SV) by high-coverage whole-genome resequencing data, and selected teat number at birth and functional teat number as two types of teat number traits for genome-wide association study (GWAS) to reveal candidate genes associated with pig teat number traits.

**Results:**

In this study, we used whole genome resequencing data from 560 Yorkshire sows to detect SNPs, InDels and SVs, and performed GWAS for the traits of born teat number and functional teat number, and detected a total of 85 significant variants and screened 214 candidate genes, including *HEG1*,* XYLT1*,* SULF1*,* MUC13*,* VRTN*,* RAP1A and NPVF.* Among them, *HEG1* and *XYLT1* were the new candidate genes in this study. The co-screening and population validation of multiple traits suggested that *HEG1* may have a critical effect on the born teat number.

**Conclusion:**

Our study shows that more candidate genes associated with pig teat number traits can be identified by GWAS with different variant types. Through large population validation, we found that *HEG1* may be a new key candidate gene affecting pig teat number traits. In conclusion, the results of this study provide new information for exploring the genetic mechanisms affecting pig teat number traits and genetic improvement of pigs.

**Supplementary Information:**

The online version contains supplementary material available at 10.1186/s12864-024-11109-0.

## Introduction

As a reproductive trait, pig teat number trait determines the lactation performance of sows, and is an important target for genetic improvement of breeding pigs [1]. Higher born teat number help sows develop more functional teats as adult. Functional teat number is one of the limiting factors in improving sows’ lactation capacity [2]. The poverty teat number of sows, underdeveloped teats and uneven arrangement of teats in the herd will reduce the piglet survival rate the economic efficiency of the farm [3]. Therefore, it is necessary to increase the number of teats in sows through genetic improvement as a way to increase the lactation capacity of sows [4].

The pig teat number trait is regulated by multiple genes [5]. With the continuous maturation of sequencing technology, genome-wide association analysis has become an important tool for screening variants related to economic traits in livestock and poultry. In recent years, GWAS has been applied to a wide range of applications in pigs, and key genes related to important economic traits such as reproduction, growth and meat quality have been identified [6–8]. The genetic variants and genes associated with pig teat number have also been extensively studied [5, 9]. So far, a total of 977 QTLs associated with the pig teat number trait have been included in the PigQTL database (https://www.animalgenome.org/cgi-bin/QTLdb/SS/browse, 2024-2-5, interview). Genes such as *VRTN* [10], *TOX3* [11], *ABCD4* [12] have been identified as candidate genes for the teat number traits. Signaling pathways related to teat or mammary gland development have also been reported, such as the Wnt signaling pathway, which affects mammary gland differentiation and determines mammary gland primordia [13]. The *TBX3* signaling pathway, which regulates mammary lineage [14], and the FGF pathway, which is closely related to mammary gland cells [15]. It is clear that identifying more genes will help us to deepen our understanding of the genetic mechanisms associated with the teat number trait. This could lead to a more appropriate breeding program to optimize the reproductive performance of the pig.

With the reduction of sequencing costs and the development of structural variant detection algorithms in recent years, GWAS based on structural variants have begun to be applied. Compared with SNPs, structural variants can carry richer genetic information due to longer sequences, and the explained heritability is higher than that of SNPs [16]. In a GWAS for porcine growth traits using SNP, InDel, SVs and tandem repeat (TR), Blaj et al. found that despite physical co-localization. SNPs or InDels did not always capture the effects of SVs and TRs on complex traits [17]. Zong et al. constructed a high-quality SV map using high-coverage sequencing data on a population of cross-bred Eurasian pigs, and performed GWAS for 25 carcass traits, 7 skeletal traits, and 4 meat quality traits, identifying that SV may affect pig body size between European and Chinese pig breeds [18]. In previous breeding work on pig teat number traits, most of the GWAS were performed using chip data, and resolution of teat number traits using resequencing data is rare.

In this context, to screen for candidate genes associated with teat number traits, this study utilized whole genome resequencing data with an average depth of more than 15× and obtained high-quality SNP, InDel and SV maps using different typing software. We then performed GWAS for born teat number and functional teat number, and finally validated the candidate genes affecting teat number traits. In conclusion, this study will provide a theoretical basis for the development of assisted breeding using SV as a molecular marker to further promote the genetic improvement of pig teat number traits in breeding.

## Materials and methods

### Animals and phenotypes

In this study, the selected Danish Yorkshire pigs were reared in the Shaanxi Shunxin Danish Breeding Pig Farm (Hanzhong City, Shaanxi Province, China). For 49,818 pigs born at the farm from 2016 to 2022, teat number related traits were collected. Including the born left teat number (LTN), the born right teat number (RTN), and the born total teat number (TTN). We also collected the date of birth, parity, sex, place of birth, and pedigree information. We traced back 3 generations of pedigree and kept 50,001 pigs in pedigree records for the next step. From July to November 2022, traits related to the number of functional teats in 430 pregnant sows on the farm were measured and recorded. We excluded undeveloped teats, damaged teats, and degraded teats, and retained the record of functional left teat number (FLTN), functional right teat number (FRTN) and functional total teat number (FTTN), as well as the information on the gestation parity, age and pedigree of the sows. Subsequently, the phenotypic data were cleaned and the abnormal values and phenotypically missing individuals were excluded for subsequent analysis. The maximum, minimum, mean, standard deviation and coefficient of variation of each trait were calculated.

### Estimation of genetic parameters

To explore the level of heritability of teat number traits in this population, genetic parameters were estimated to calculate heritability of teat number traits in Yorkshire pigs by the maternal effect model using ASReml (version 4.1.0) software. The model was used for the born teat number traits as follows:

$$\:{\varvec{Y}}_{1}=\varvec{X}{\varvec{b}}_{1}+{\varvec{Z}}_{1}\varvec{a}+{\varvec{Z}}_{2}\varvec{c}+\varvec{e}$$

Where the $\:{\varvec{Y}}_{1}$ is the vector of born teat number phenotypes, $\:{\varvec{b}}_{1}$ fixed effects vector includes pig farm-year-season, parity, and sex effects, $\:\varvec{a}$ is the vector of individual additive effects, $\:\varvec{c}$ is the vector of maternal environmental effects, $\:\varvec{e}$ is the residual effects, and $\:\varvec{X}$, $\:{\varvec{Z}}_{1}$ and $\:{\varvec{Z}}_{2}$ are the corresponding structure matrices.

For the functional teat number traits, the model was used as follows:

$$\:{\varvec{Y}}_{2}=\varvec{X}{\varvec{b}}_{2}+{\varvec{Z}}_{1}\varvec{a}+{\varvec{Z}}_{2}\varvec{c}+\varvec{e}$$

Where $\:{\varvec{Y}}_{2}$ is the vector of functional teat number phenotypes, $\:{\varvec{b}}_{2}$ is a vector of fixed effects including sow parity of pregnancy and age effects, $\:\varvec{a}$ is an individual additive effects vector, $\:\varvec{c}$ is the vector of maternal environmental effects, $\:\varvec{e}$ is the residual effects, and $\:\varvec{X}$, $\:{\varvec{Z}}_{1}$ and $\:{\varvec{Z}}_{2}$ are the corresponding structure matrices.

To explore potential relationships prior to the traits of born teat number and functional teat number, we calculated genetic correlations between traits using ASReml (version 4.1.0) and estimated the heritability for 430 sows with same traits.

### Genotypic data and quality control

In this study, whole genome resequencing data were collected from 560 pig samples that had published in our previous studies (Sun et al. Unpublished). Briefly, after collecting ear tissue samples from 560 Yorkshire sows, DNA was extracted and whole-genome resequencing was performed using the DNBSEQ-T7 platform, yielding sequencing data with an average sequencing depth of 15.6×. We processed the raw data to remove low-quality reads using the default parameters of Fastp (version 0.20.1) [19]. Then, the clean reads were aligned to the *Sscrofa11.1* (https://www.ncbi.nlm.nih.gov/) reference genome from NCBI using BWA (version 0.7.15) (BWA-MEM algorithm, default parameters) [20]. And we obtained the clean Binary Alignment Map format (bam) files by removing duplicate reads and sorting them. Subsequently, these files were used to generate the gvcf file using the HaplotypeCaller program in the GATK (version 4.1.1) pipeline [21]. Then the gvcf files were merged by the GenomicsDBImport module. Finally, GenotypeGVCFs was used to convert database into a vcf file. We filtered the variant data-sets of SNP and InDel (Insertion and deletion,2–50 bp), using the best practice parameters “QUAL < 30.0 | QD < 2.0 | MQ < 40.0 | FS > 60.0 | SOR > 3.0 | MQRankSum < -12.5 | ReadPosRankSum < -8.0” and “QD < 2.0 | QUAL < 30.0 | FS > 200.0 | ReadPosRankSum < -20.0”. We then retained biallelic SNPs and InDels.

**Fig. 1:**
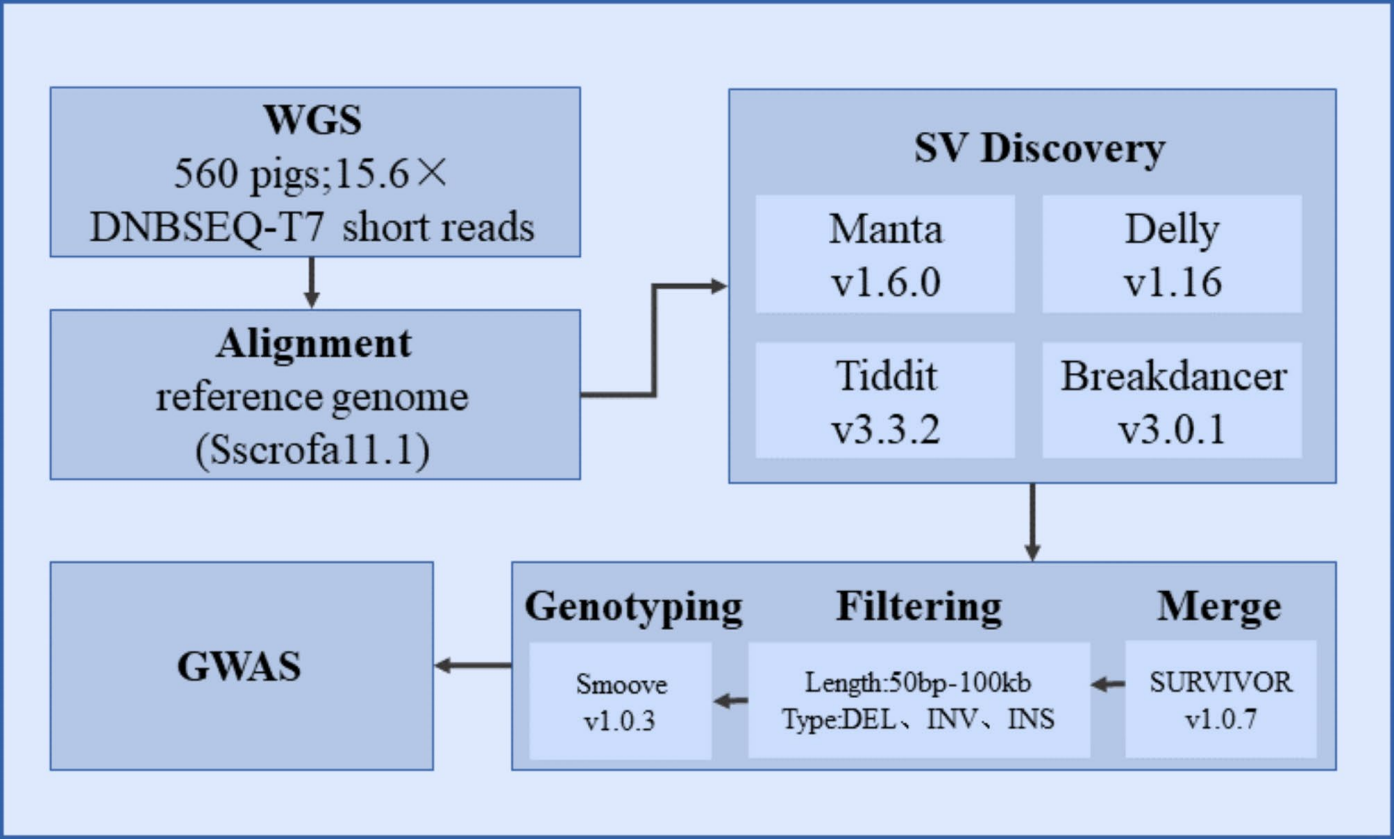
SV discovery process overview

In terms of SV detection, we used four software, including Manta (version 1.6.0) [22], Delly (version 1.16) [23], Tiddit (version 3.3.2) [24] and Breakdancer (version 3.0.1 ) [25], to detect the SVs from 560 pig samples, and then we used SURVIVOR (version 1.0.3) [26] to merge the SVs site that were called by at least two tools. Finally we used Smoove (version 0.2.8) [27] software to genotype SV type and used Bcftools (version 1.10.2) [28] to merge sample SV genotype files(Fig. 1).

SNPs sets: PLINK (version 1.9.0) [29] was used for quality control, based on the following exclusion criteria: SNPs with miss genotype call rate > 0.1, samples with miss genotype call rate > 0.1, minor allele frequency(MAF) < 0.01, and Hardy-Weinberg equilibrium *P* < 1 × 10^− 5^, and using the “--indep-pairwise 50 5 0.5” parameter to prune SNPs by linkage-disequilibrium(LD). Finally, 18 autosomal 678,204 SNPs were retained for further analysis.

InDels sets: PLINK (version 1.9.0) was also utilized for quality control with the parameters of “--gene 0.01 –maf 0.05”, and the “--indep-pairwise 50 5 0.1” was used to prune InDels by LD. A total of 560 samples and 103,491 variants in 18 autosomes were retained for GWAS.

SVs sets: SV lengths were filtered to retain SVs within the range of 50 bp-100,000 bp, and only three types of SVs, INV, INS and DEL, were retained, and a total of 12,671 SVs from 18 autosomes were finally used for GWAS.

### Genome-wide association analysis for each trait

To efficiently detect the variation associated with the low heritability teat number trait in this study, we used the Bayesian-information and Linkage-disequilibrium Iteratively Nested Keyway (BLINK) for GWAS by GAPIT (version 3.0) [30]. The BLINK model used the phenotype of each teat trait as the response variables and the numeric variants matrix as the genetic data. The significance threshold line 0.05/N (N is the number of variants) and the potential threshold line 1/N in the results of GWAS were determined by Bonferroni-corrected, and the variant data included SNP, InDel, and SV obtained after quality control. We used the R package CMplot (version 4.2.0) to gain the Manhattan plots and QQ-plots [31].

### Candidate gene search and functional enrichment analysis

We searched the candidate genes which including or close to significant variants using BedTools (version 2.27.1) [32] based on *Sscrofa11.1*. The screening range was 0.5 Mb region upstream and downstream of the significant genetic variants. Subsequently, we used the gProfiler [33] website (https://biit.cs.ut.ee/gprofiler/gost) to enrich and analyze the candidate genes to reveal the functions of the candidate genes and the involvement of the candidate genes through Gene Ontology (GO)) and Kyoto Encyclopedia of Genes and Genomes (KEGG) pathways.

In order to obtain genes screened together by different variant types, we selected the intersection of candidate genes screened by SNPs, InDels and SVs, and then used R package to draw the Venn map. LDBlockShow (version 2.6) [34] was used to generate and visualize the local linkage disequilibrium (LD) pattern of haplotype block between significant variants and genes.

### Population validation of candidate genes

For the same candidate genes screened multiple times by GWAS, we selected 1,169 Yorkshire sows from another farm, collected their born total teat number, and genotyped them by target sequencing chip (GenoBaits Porcine 50 K Chip). We found the variant located in the candidate gene region in the validation population, extracted the variant and genotype of them for significance test with the phenotype of the validation population, and confirmed their significance by rank sum test.

## Result

### Descriptive statistics of phenotype and heritability

Descriptive statistics of the phenotypes of born teat number traits and functional teat number traits of Danish Yorkshire sows collected in this study are shown in Table 1. We found that the number of teats traits in pigs was a low heritability trait with heritabilities ranging from 0.085 to 0.173, while their coefficient of variation was less than 10%. The genetic correlation between traits were appearing positive correlation. Although the heritability of the born teat number traits and functional teat number traits were different in shared trait population (*N* = 430), both of them were at low heritability level (Table 2).

**Table 1:** Descriptive statistics of traits

Trait	*N*	Max	Min	Mean (SD)	CV (%)	h^2^
LTN	49,752	9	6	7.059 ± 0.283	4.007	0.104
RTN	49,714	9	6	7.175 ± 0.409	5.706	0.168
TTN	49,664	18	12	14.235 ± 0.581	4.083	0.173
FLTN	427	8	6	7.019 ± 0.464	6.615	0.085
FRTN	430	9	6	7.114 ± 0.532	7.478	0.112
FTTN	427	18	12	14.138 ± 0.800	5.661	0.151

N: number of samples; Max: maximum value; Min: minimum value; Mean: arithmetic mean; SD: standard deviation; CV: coefficient of variation; *h*^2^: heritability

**Table 2:** The genetic correlation between teat number traits

Trait	LTN	RTN	TTN	FLTN	FRTN	FTTN
LTN	0.005	0.779	0.905	0.937	0.738	0.916
RTN		0.253	0.972	0.971	0.912	0.983
TTN			0.005	0.794	0.890	0.950
FLTN				0.085	0.721	0.858
FRTN					0.112	0.970
FTTN						0.151

Genetic correlation above the diagonal. Heritability on the diagonal

### Genome-wide association analysis for the born teat number traits

**Fig. 2:**
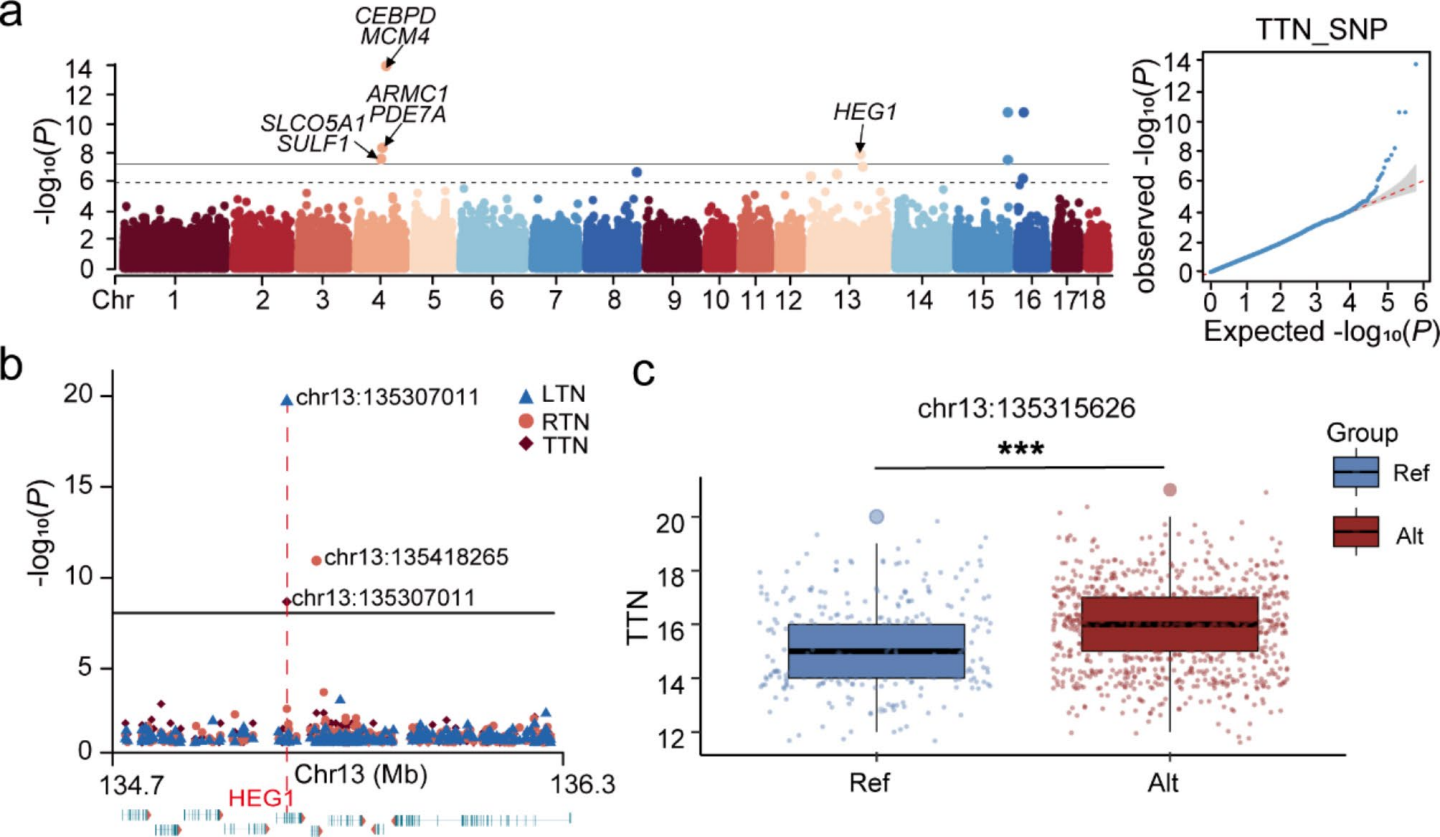
GWAS of born teat number based on SNPs and population validation. **(a)** GWAS of TTN based on SNPs. *N* = 560 Yorkshire pigs. The horizontal solid line represents Bonferroni threshold (-Log_10_(0.05/678,204) = 7.1324). The horizontal dashed line represents the suggestive significance threshold line (-Log_10_(1/678,204) = 5.8314). **(b)** Magnification of the gene region with significant variants in TTN, RTN and LTN GWAS result. Different color and shaped points distinguish three traits. **(c)** Population validation in 1169 Yorkshire pigs base on target sequencing with GWAS candidate significant SNP in TTN. The blue box represent sow population with reference variant; and the red box is sow population with mutation

In this study, 560 Yorkshire sows were used for GWAS of born teat number traits and 27, 26, and 20 significant variants were detected in the GWAS results for SNP, InDel, and SV, respectively. This significant SNPs were distributed on several chromosomes, e.g., Chr3, Chr4, Chr12, Chr13(Fig. 2a). Within Chr13, 134.7 Mb-136.3 Mb, nine genes were identified to be associated with born teat number traits, including *HEG1*, *MUC13*, *UMPS*, *OSBPL11*, *SNX4*, *ZNF148*, *SLC12A8*, *ITGB5*, and *KALRN*. The significant SNP chr13:135307011 was screened for both LTN and TTN, and this significant SNP was located in an exon of the *HEG1* gene (Fig. 2b). Interestingly, the *HEG1* gene was also screened within 60Kb upstream of the significant SNP chr13:135418265, which affects the number of teats on the right side. After enrichment analysis, *HEG1* was enriched for epithelium development, which is strongly associated with mammary gland development. To validate the effect of *HEG1* on teat number in a sow population, we evaluated the effect of variation on this gene on total teat number in target sequencing from another Yorkshire sow population (*n* = 1169), and found that mutations in *HEG1* gene region were significantly associated with born total teat number (*P* < 0.001) in this population (Fig. 2c). The candidate genes of TTN by annotation are listed in Table 3.

**Table 3:** Significant SNPs for TTN

Traits	SNPs	Pos (bp)	*P*-value	Candidate Gene
TTN	chr4:65608738	65,608,738	3.56 × 10^− 8^	*PRDM14*,* SLCO5A1*,* SULF1*,* NCOA2*
	chr4:69022640	69,022,640	6.31 × 10^− 9^	*TRIM55*,* ARMC1*,* PDE7A*
	chr4:79455541	79,455,541	1.70 × 10^− 14^	*CEBPD*,* MCM4*,* SPIDR*,* SNTG1*,* PRKDC*
	chr13:135307011	135,307,011	1.77 × 10^− 8^	*HEG1*,* MUC13*,* OSBPL11*,* SLC12A8*,* ITGB5*,* KALRN*
	chr15:136826587	136,826,587	2.63 × 10^− 11^	*PRLH*
	chr15:136853009	136,853,009	4.19 × 10^− 8^	*PRLH*
	chr16:16346505	16,346,505	2.65 × 10^− 11^	——

**Fig. 3:**
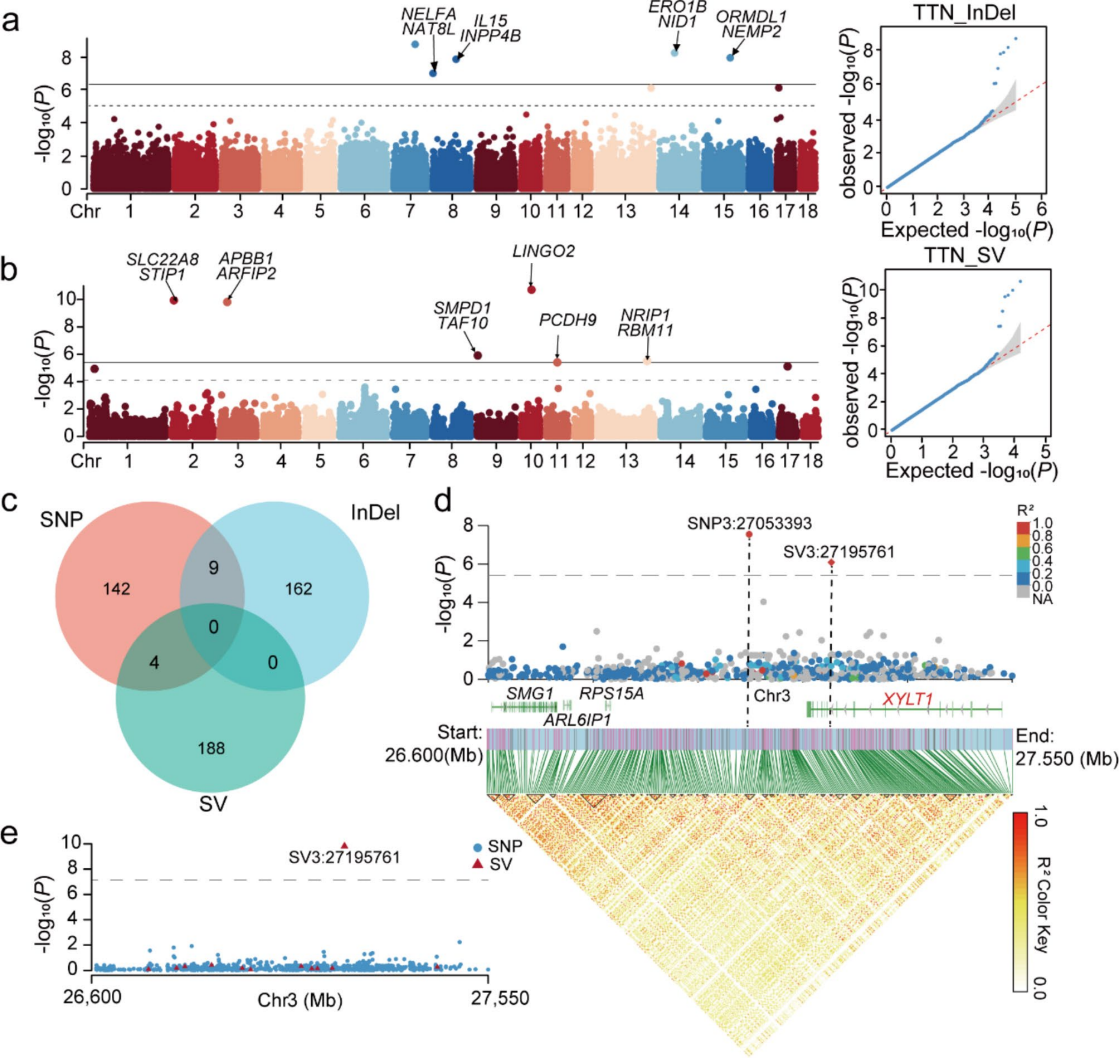
GWAS of born teat number traits. **(a)** TTN GWAS base on InDels. **(b)** TTN GWAS base on SVs. **(c)** Candidate genes from GWAS screens for SNPs, Indels, and SVs, respectively, plotted on a Wayne diagram. **(d)** Magnified Manhattan plot of LTN GWAS result and LD heatmap of the magnified region. **(e)** Magnified Manhattan plot of GWAS result in TTN

A total of 5 significant InDels and 10 significant SVs were detected in TTN (Fig. 3a and b). In addition, a total of 16 significant InDels and 14 significant SVs, and 5 significant InDels and 3 significant SVs were detected in RTN and LTN, respectively (Fig. S2). The information of significant InDels, SVs and the genes obtained after annotation are listed in Table S1 and Table S2.

For GWAS of born teat number traits, the genes obtained based on the annotation of different types of genetic variants were taken to the intersection (Fig. 3c). We found that SNP and InDel co-screened nine genes, namely *HEG1*, *ITFGB5*, *KALRN*, *MUC13*, *OSBPL11*, *SLC12A8*, *SNX4*, *UMPS*, and *ZNF148*. And 17 genes, including *ARL6IP1*, *RPS15A*, *SMG1*, *XYLT1* and others, were screened together in GWAS based on SNP and SV. Within the 26.600 Mb-27.550 Mb region of chromosome 3, we found that the significant SV3:27195761 was located within the *XYLT1* gene region, while SNP3:27053393, although already adjacent to *XYLT1*, still did not directly detect significant variants within the gene region base on SNP GWAS in LTN (Fig. 3d). Compared with SV3:27195761, SNP3:27053393 is more distant from *XYLT1*. In the analysis of TTN trait, we also screened the SV3:27195761 hotspot and annotated *ARL6IP1*, *RPS15A*, *SMG1*, *XYLT1*(Fig. 3e). However, the GWAS based on SNP of TTN did not find this hotspot. So, GWAS based on SV has more potential to capture the target genes.

### Genome-wide association analysis for the functional teat number traits

**Fig. 4:**
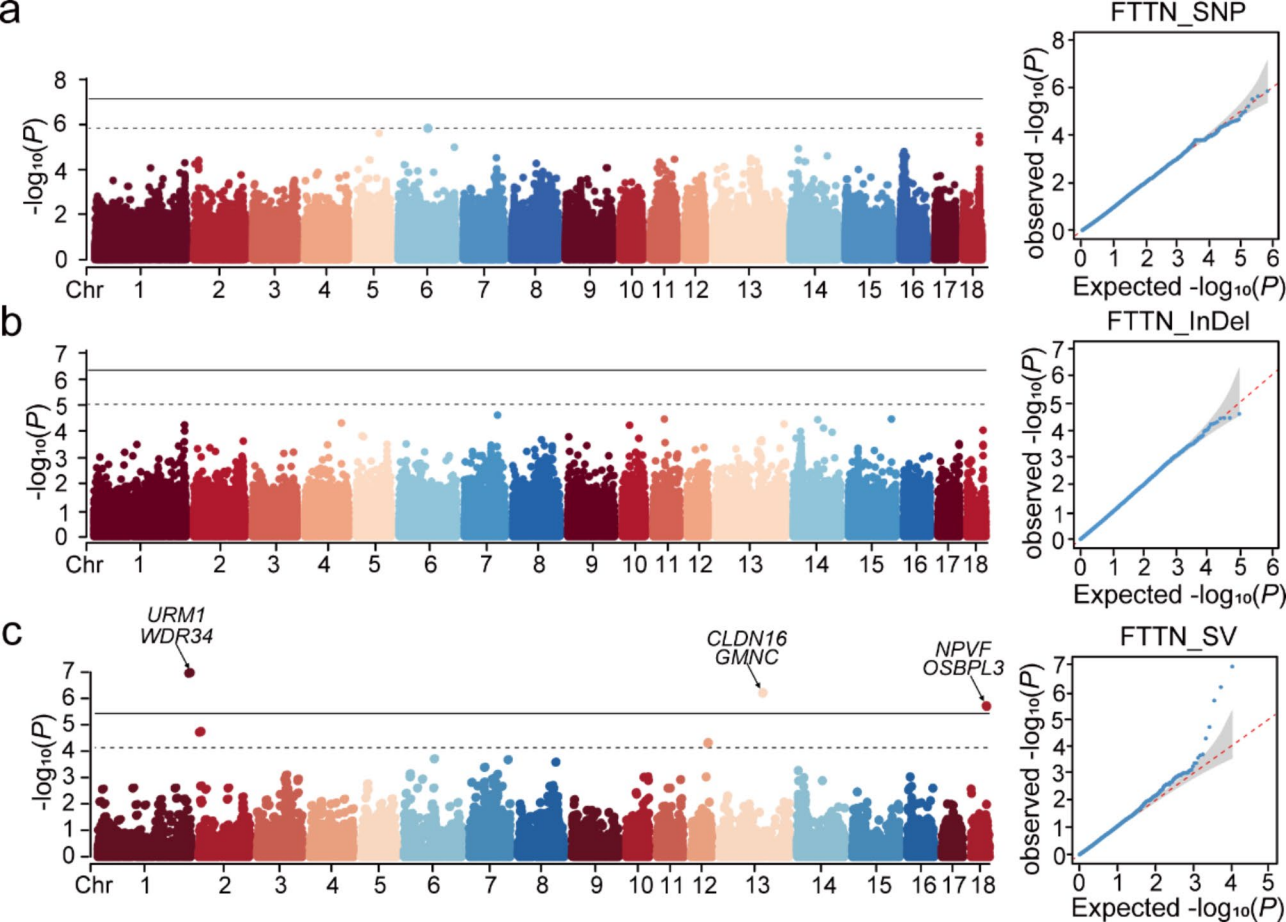
GWAS of FTTN base on SNPs, InDels, SVs, respectively. **(a)** GWAS result of FTTN base on SNPs. *N* = 427 pig samples. **(b)** GWAS result of FTTN base on InDels. **(c)** GWAS result of FTTN base on SVs

The functional teat number of sows determines the ability of lactation, and the number of functional teats is closely related to the survival rate of lactating piglets. Therefore, we collected functional teat number trait records from 430 sows and performed GWAS combining three types variant after removing outlier phenotypes. Overall, the number of significant variants detected by GWAS for functional teat number was 2, 3, and 7 for SNPs, InDels, and SVs, respectively. FTTN was not screened for genome-wide significant SNPs and InDels (Fig. 4a and b). But SV-based GWAS identified three significant regions, annotated to genes such as *NPVF*, *SWI5*, *URM1*(Fig. 4c; Table 4).

**Table 4:** Significant SVs for functional teat number relevant traits

Traits	Chr	Start	End	Type	*P*-value	Candidate Gene
FTTN	1	268,509,186	268,509,485	DEL	1.11 × 10^− 7^	*LCN2*,* ODF2*,* SWI5*,* TRUB2*,* URM1*,* WDR34*
	13	127,891,501	127,891,579	DEL	6.32 × 10^− 7^	*CLDN1*,* CLDN16*,* P3H2*,* SDK1*,* GMNC*
	18	47,349,893	47,350,429	DEL	2.01 × 10^− 6^	*NPVF*,* OSBPL3*

**Fig. 5:**
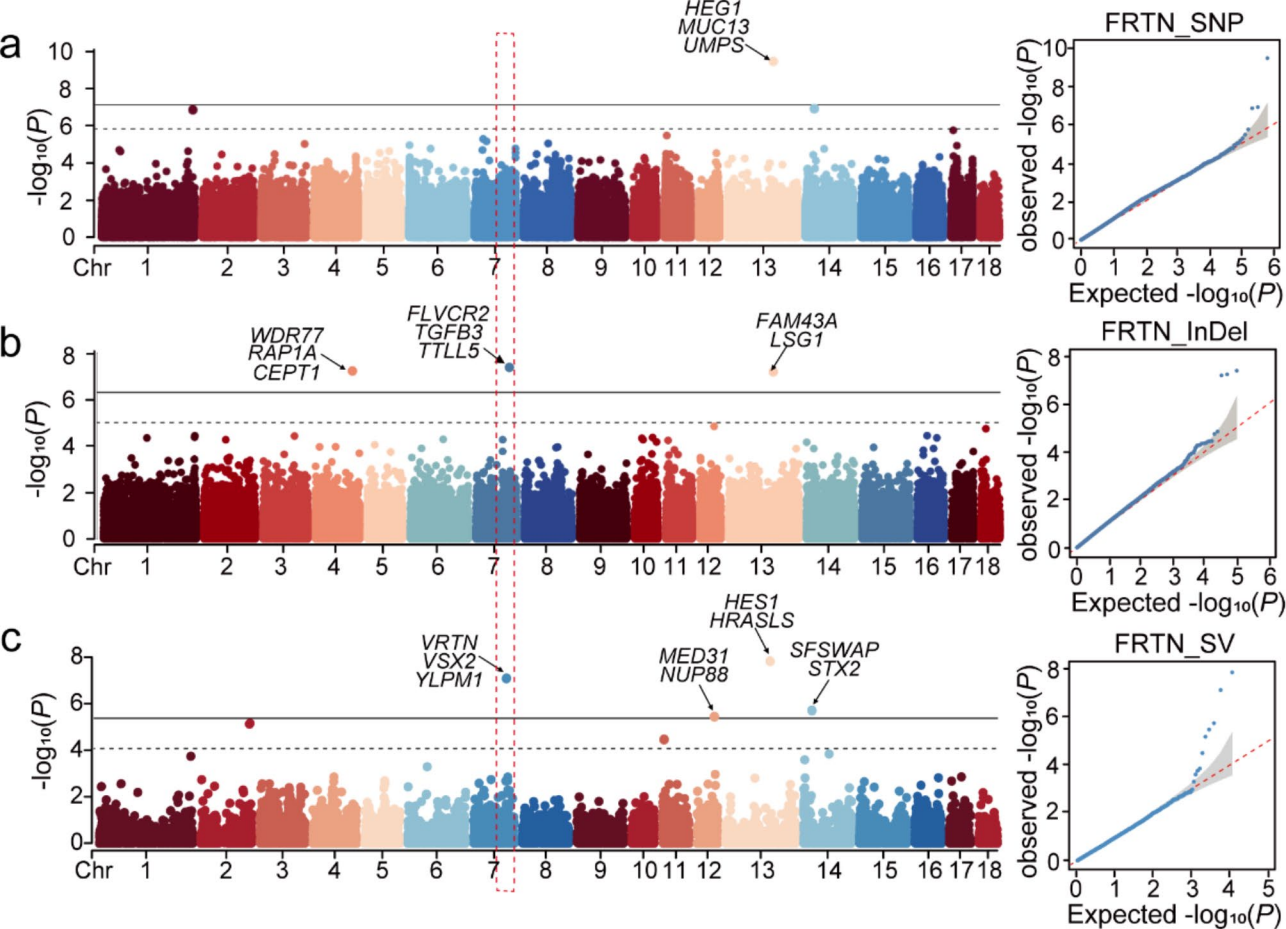
GWAS of FRTN base on SNPs, InDels, SVs, respectively. *N* = 430 pig samples. The dotted red box indicates that the same region of interest was detected by the three variant types GWAS results in Chr7. **(a)** GWAS of FRTN base on SNPs. There is no significant variation on Chr7, but trends can be detected. **(b)** GWAS of FRTN base on InDels. **(c)** GWAS of FRTN base on SVs

We identified *VRTN* in the SV-based GWAS for FRTN trait (Fig. 5c; Table 5), and *VRTN* has been reported in several studies related to pig teat number [35]. Although this gene was not identified in the SNP and InDel-based GWAS, we could see a clear peak trend on chromosome 7 (Fig. 5a and b, Table S4, Table S5). For new candidate genes, *RAP1A* was a potential gene of functional teat number, which was detected by InDel-based GWAS of FRTN. We screened only one significant SNP locus associated with FLTN trait, annotated to genes such as *SLC4A7* and *NEK10*, and unfortunately, no relevant genetic markers were detected based on InDel and SV (Fig. S3, Table S4).

**Table 5:** Significant SVs for functional right teat number trait

Traits	Chr	Start	End	Type	*P*-value	Candidate Gene
FRTN	7	97,757,361	97,757,668	DEL	7.76 × 10^− 8^	*ABCD4*,* ALDH6A1*,* AREL1*,* COQ6*,* EIF2B2*,* FAM161B*,* FCF1*,* LIN52*,* LTBP2*,* NPC2*,* PROX2*,* SYNDIG1L*,* VRTN*,* YLPM1*,* VSX2*
	12	51,093,256	51,093,314	DEL	3.44 × 10^− 6^	*MIS12*,* WSCD1*,* NUP88*
	13	130,756,600	130,756,898	DEL	1.42 × 10^− 8^	*HES1*,* HRASLS*
	14	24,058,687	24,059,355	DEL	1.86 × 10^− 6^	*ADGRD1*,* SFSWAP*,* STX2*

### Enrichment analysis of candidate genes

Table S6 shows the results of the GO term enrichment and KEGG pathway of candidate genes. The GO term enrichment was involved in terms such as Wnt signaling pathway (GO:0016055), animal organ formation (GO:0048645), epithelium development (GO:0060429), cellular process involved in reproduction in multicellular organism (GO:0022412), regulation of developmental process (GO:0050793). Furthermore, these candidate genes are involved in the cG/MP-PKG signaling pathway (KEGG:04022), JAK-STAT signaling pathway (KEGG:04630), PI3K-Akt signaling pathway (KEGG:04151) and other pathways.

## Discussion

Sow teat number affects the survival rate of lactating piglets and is an important indicator of lactation ability, and increasing the teat number can improve sow reproductive performance [36, 37]. Related studies have shown that there is genetic variation in the teat number and some of the reproductive traits of sows, which has a profound effect on the weight of piglets 10 days after birth [38, 39]. When sows do not have enough teats to satisfy piglets’ nursing needs, producers opt for fostering methods or even euthanize piglets that are not viable [40]. There are many factors that influence teat traits and there are numerous research reports on them. There are inter- and intra-breed differences in pig teat numbers, and generally breeds with high reproductive performance have relatively high teat numbers [41]. The hormone levels of the animal’s organism will determine the development of mammary glands and the formation of teats, such as growth hormone, prolactin, estrogen, etc [42–44].

In this study, we collected far more the born teat number traits records than the functional teat number traits due to the varying ease of phenotype collection. Although the genetic correlation between the functional teat number traits and the born teat number traits suggests a similar mechanism of inheritance for both traits, the heritability of the traits in the shared traits population also implies that it may have genetic differences between the born teat number traits and the functional teat number traits.

For the TNN trait, we identified *SULF1*, *SLCO5A1* and *NCOA2* candidate genes on chromosome 4. It has been shown that these genes are the candidate genes of rib number trait in pigs, and the number of ribs determines the body length of pigs [45]. Therefore, *SULF1*, *SLCO5A1*, and *NCOA2* genes may be involved in the growth and development process of pigs, which affects the number of ribs and increases the body length, thereby favoring the increase in teat number. After conducting GWAS based on SNP for TTN, RTN and LTN traits, we found that these results co-localized with the *HEG1* gene. Interestingly, we also identified this gene in an InDel-based GWAS for the RTN trait. *HEG1* has previously been reported to be involved in the regulation of the Wnt signaling pathway [46], which is closely associated with mammary gland development. Moreover, *HEG1* is enriched in epithelium development, and teat development is also similarly regulated by this type of pathway [47]. And we found variant typing within the *HEG1* region in chip data from another Yorkshire sow population and when the locus in this region was mutated, the number of teats in the population was significantly higher than in sows that were not mutated. Therefore, the *HEG1* gene may be a key candidate gene affecting the pig teat number trait, and its specific molecular mechanism needs to be further explored.

It has been shown that combining SNP, InDel and SV explains higher heritability of traits and improves the power to identify genetic factors underlying complex traits [16]. In this study, we co-located a candidate gene *XYLT1* by GWAS based on SNP and SV in the LTN trait. This gene has been associated with systemic wrinkle in the aromatic pig in previous studies [48], and we hypothesized that it is likely that this gene also affects embryonic teat formation; it has also been suggested that *XYLT1* is a key regulator of bone length [49], and considering that many proteins that regulate mammary gland development have dual roles in regulating both mammary gland development and bone development [50], we suggest that *XYLT1* is a key candidate gene associated with the pig teat number trait. We also identified the *ACP1* gene, which has been reported to be involved in growth factor signaling in mammary gland development and is associated with inverted teats in pig [51]. The *AHCTF1* gene determines live piglet production traits [52], and a high number of teats prevents piglets from competing for teats due to insufficient teats. *CTBP1* has previously been reported to be involved in mammary gland development in mice [53]. *TRIML1*, *TRIML2* and *ZFP42* genes were identified in a study of teat number in local pigs [54]. *RPS15A* was screened by two variants together, and it was reported that this gene is closely related to mammary gland development and is also expressed in porcine mammary tissue [55].

Functional teat number is a key factor in determining the lactation power of sows. In the GWAS for FTTN, we did not screen for significant genetic markers. There are two possible reasons for this: on the one hand, the phenotype may have a skewed in distribution due to the small sample size (*N* = 427), and on the other hand, it may be that the trait of functional teat number itself is more sensitive to the environment, leading to the mixing of noise in the process of performing the GWAS. However, we found a clear peak trend on chromosome 7 in the FRTN analysis. We also identified the *VRTN* gene, which has been repeatedly reported to be associated with pig teat number [11, 56]. On effective teat number, we also identified the *HEG1* gene, which is not only involved in the Wnt signaling pathway, but also associated with cardiovascular development [46, 57]. Therefore, we speculate that the *HEG1* gene may play a role in the vascularization of the mammary gland, which affects the vascularization, and when the mammary gland is underdeveloped, the phenomenon of blind teat and inverted teat occurs in production, which indirectly contributes to the number of effective teats.

Among other genes of our interest, *RAP1A* is essential for epithelial acinar structure and lumen formation in the human breast [58], and it is required for angiogenesis [59]. *NPVF* is a candidate gene capable of regulating reproduction, which could regulate breeding season of mare [60], and it could also regulate hypothalamic hormone expression. So, we considered that *NPVF* is a new candidate gene of functional teat number traits. The *SLC4A7* gene has been previously reported to be a good candidate for the intramuscular fat content of the Beijing black pig [61], which determines fat deposition in cattle and is associated with backfat thickness [62]. Milk is rich in nutrients, and regulation of *SLC4A7* gene would improve milk composition and contribute to the health of lactating piglets. The *CHIA* gene is highly expressed in udder tissues, which is a favorable condition for it to be a key candidate gene. *FLVCR2* has not only been reported to be associated with porcine teat number, but also as a candidate gene for porcine rib number [63, 64]. *TGFB3* expression plays a role in the growth and development of follicular membrane cell and granulosa cell growth and development, regulates the proliferative state and cholesterol homeostasis of the developing mammary gland in infancy, and also determines embryonic gonadal development, and has also been reported in breast cancer studies [65–67]. Nonetheless, we did not explore the effect of sex chromosomes on functional teat number traits, and we will delve into the trait of sex chromosomes on teat number.

## Conclusion

We performed a GWAS based on the teat number trait using three genetic variants, SNP, InDel and SV, and screened candidate genes such as *HEG1* and *XYLT1*. For the number of functional teats, we proposed two new genes related to the number of sows’ teats, *RAP1A* and *NPVF*. These genes provide theoretical references and new targets for molecular marker breeding of high-breeding sows.

## Electronic supplementary material

Below is the link to the electronic supplementary material.

Supplementary Material 1

Supplementary Material 2

Supplementary Material 3

Supplementary Material 4

## Data Availability

The variation data analysed in this paper have been deposited in the European Variation Archive (EVA) at EMBL-EBI under accession number PRJEB82721.
