# Supplementary figures and images for: Revealing genes related teat number traits via genetic variation in Yorkshire pigs based on whole-genome sequencing
Source: BMC Genomics. 2024 Dec 18;25:1217. doi: 10.1186/s12864-024-11109-0 (PMC11657392; doi:10.1186/s12864-024-11109-0)

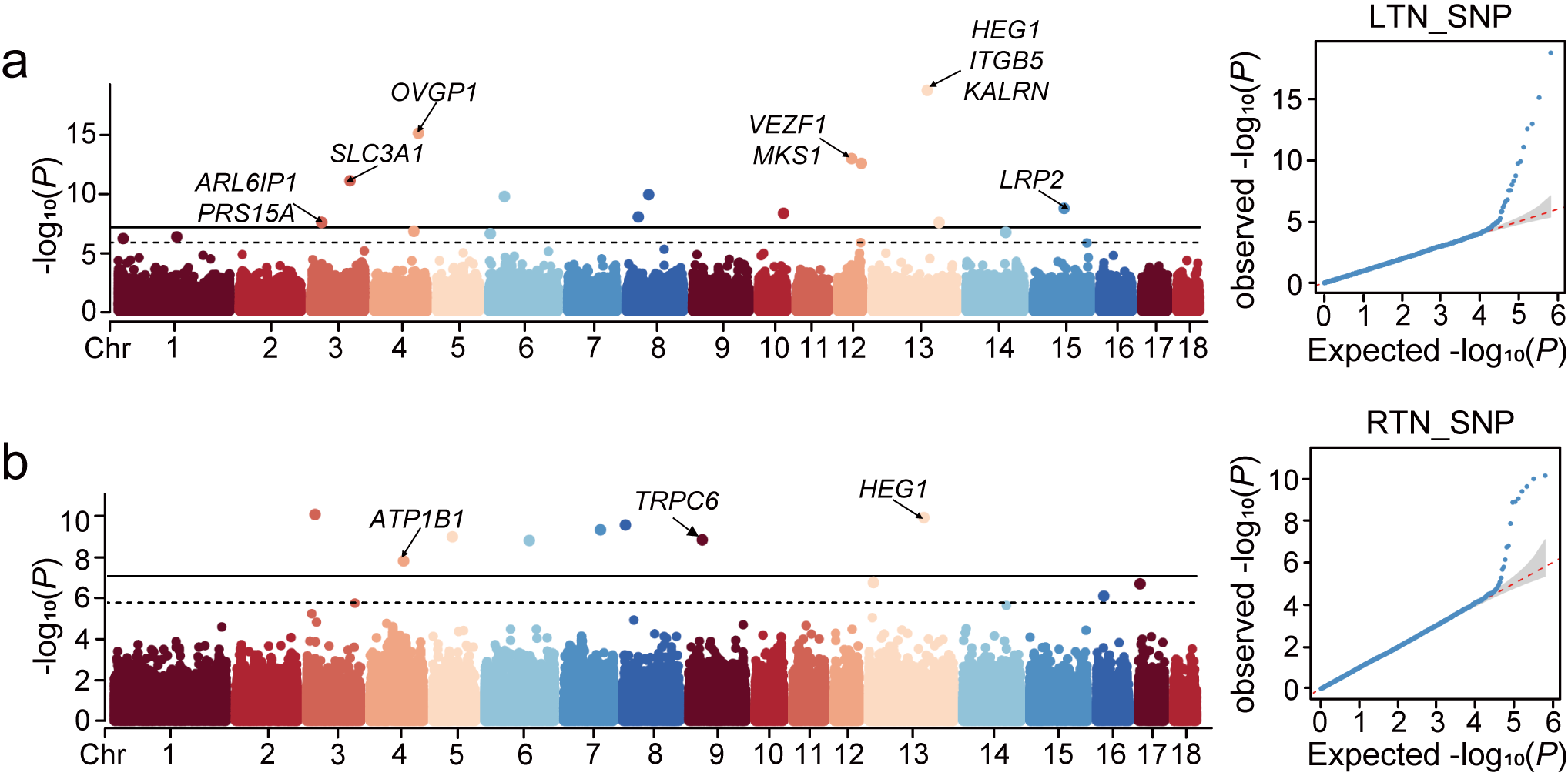

Supplement: Supplementary file 1 — Supplementary Material 1 [file 12864_2024_11109_MOESM1_ESM.png]

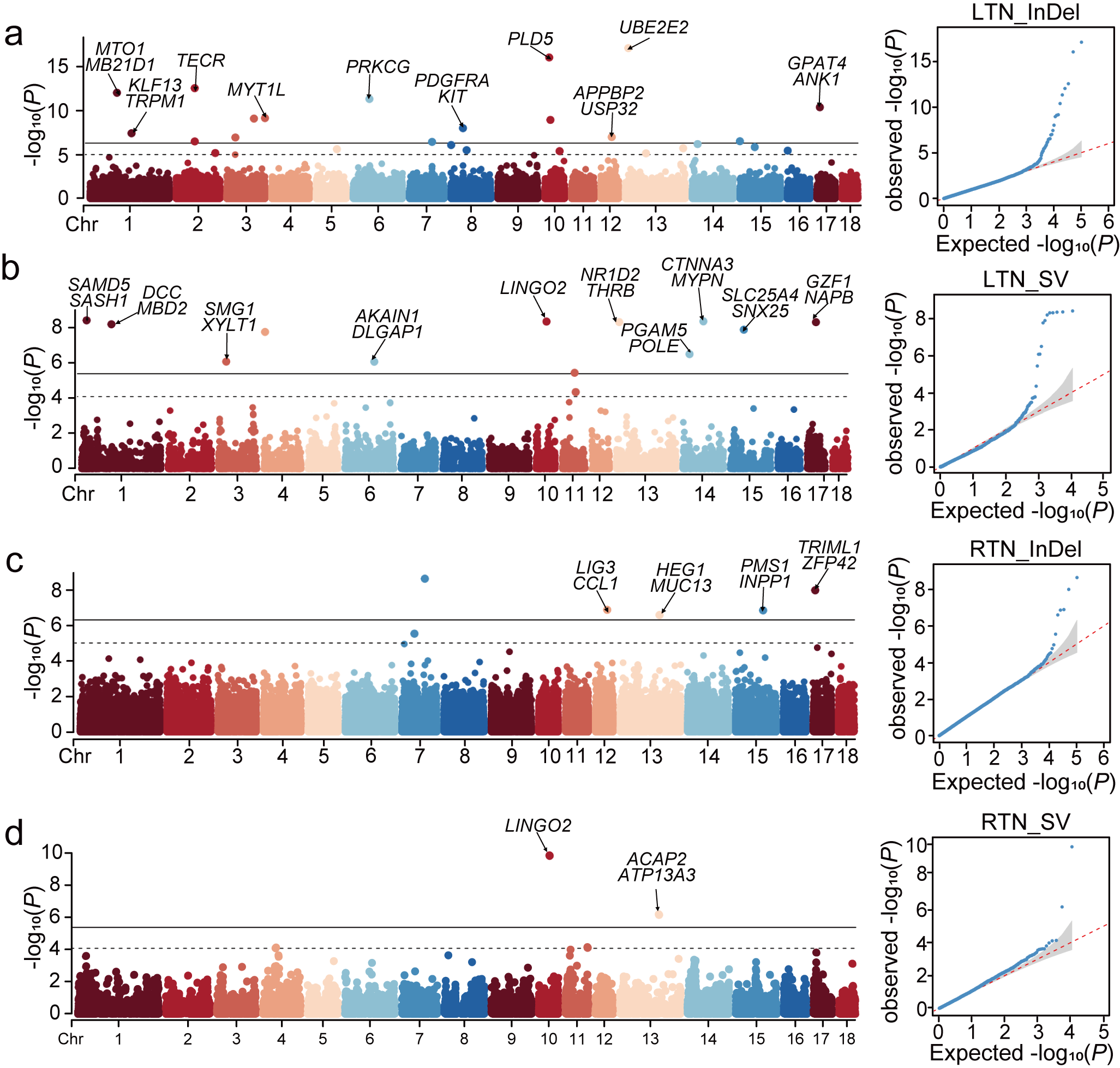

Supplement: Supplementary file 2 — Supplementary Material 2 [file 12864_2024_11109_MOESM2_ESM.png]

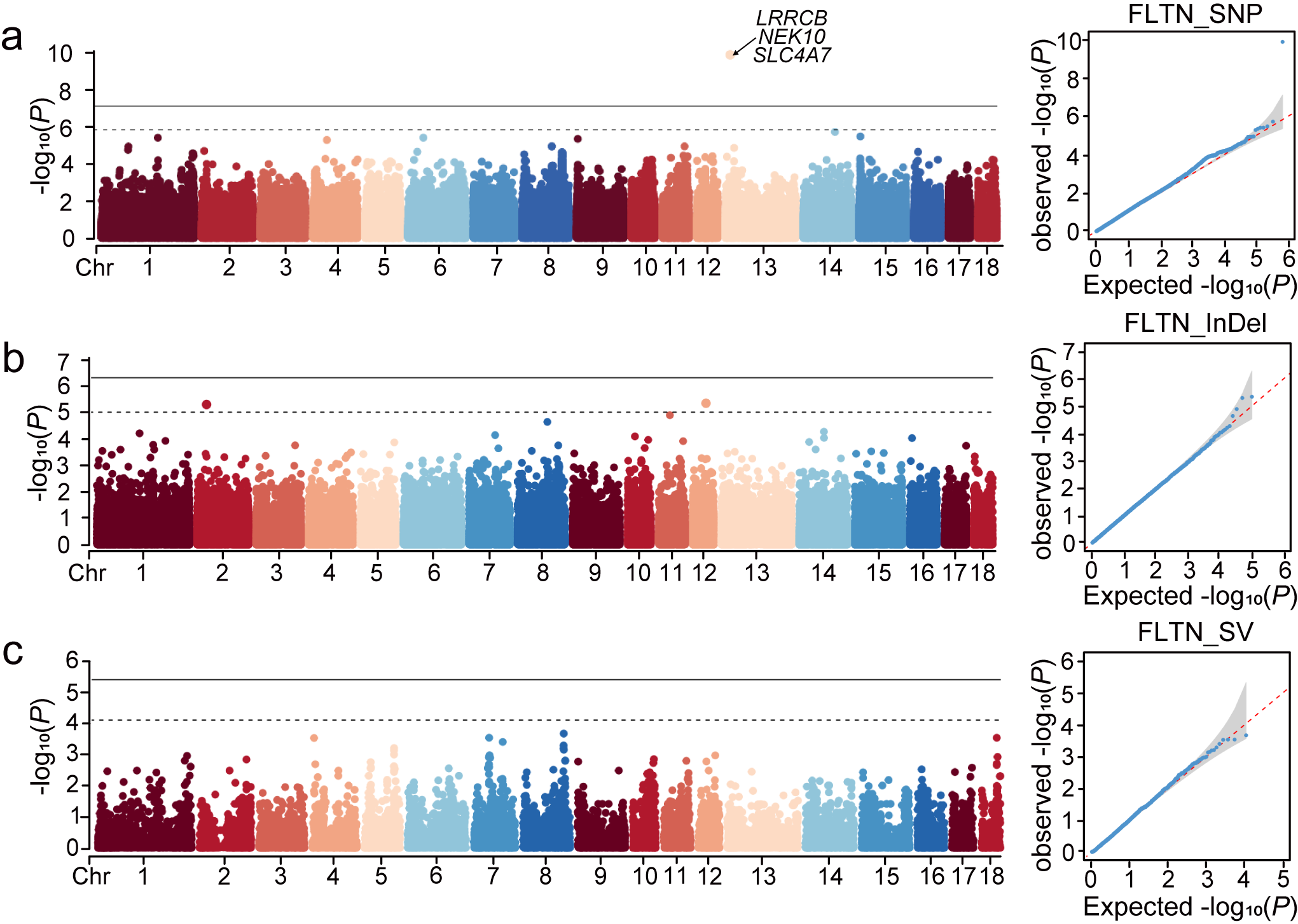

Supplement: Supplementary file 3 — Supplementary Material 3 [file 12864_2024_11109_MOESM3_ESM.png]
