# Supplementary material for: Revealing genes related teat number traits via genetic variation in Yorkshire pigs based on whole-genome sequencing
Source: BMC Genomics. 2024 Dec 18;25:1217. doi: 10.1186/s12864-024-11109-0 (PMC11657392; doi:10.1186/s12864-024-11109-0)
Supplement: Supplementary file 4 — Supplementary Material 4 [file 12864_2024_11109_MOESM4_ESM.docx]

**TableS1** Significant SNPs of born left teat number trait and born right teat number trait.

| Traits | SNPs | Pos (bp) | *P*-value | Candidate Gene |
| --- | --- | --- | --- | --- |
| LTN | chr3:27053393 | 27053393 | 2.82×10^-8^ | *ARL6IP1, RPS15A, SMG1, XYLT1* |
|  | chr3:96453060 | 96453060 | 8.29×10^-12^ | *SLC3A1, LRPPRC, ABCG5, CAMKMT, THADA* |
|  | chr4:108573951 | 108573951 | 7.62×10^-16^ | *ADORA3, OVGP1, KCND3* |
|  | chr6: 38779433 | 38779433 | 1.81×10^-10^ | —— |
|  | chr8:28825209 | 28825209 | 9.93×10^-9^ | *RELL1* |
|  | chr8:54305767 | 54305767 | 1.25×10^-10^ | *TMEM165* |
|  | chr10:61080457 | 61080457 | 4.87×10^-9^ | *CELF2* |
|  | chr12:34225319 | 34225319 | 1.08×10^-13^ | *SRSF1, MPO, MRPS23, VEZF1, MKS1, RNF43, HSF5* |
|  | chr12:58091305 | 58091305 | 2.73×10^-13^ | *HS3ST3B1, COX10* |
|  | chr13:135307011 | 135307011 | 1.70×10^-19^ | *HEG1, MUC13, OSBPL11, SLC12A8, ITGB5, KALRN* |
|  | chr13:164207551 | 164207551 | 2.88×10^-8^ | —— |
|  | chr15:75483540 | 75483540 | 1.84×10^-9^ | *NOSTRIN, G6PC2, ABCB11, DHRS9, SPC25, LRP2* |
| RTN | chr3:20412064 | 20412064 | 6.80×10^-11^ | —— |
|  | chr4:81776657 | 81776657 | 1.31×10^-8^ | *CCDC181, SLC19A2, ATP1B1* |
|  | chr5:47776412 | 47776412 | 8.51×10^-10^ | *ITPR2* |
|  | chr:108929927 | 108929927 | 1.29×10^-9^ | —— |
|  | chr:89604570 | 89604570 | 3.85×10^-10^ | —— |
|  | chr8:6248614 | 6248614 | 2.24×10^-10^ | *ZNF518B* |
|  | chr9:32920419 | 32920419 | 1.20×10^-9^ | *ANGPTL5, BIRC3, MMP27, MMP8, TRPC6, CEP126, YAP1* |
|  | chr13:135418265 | 135418265 | 9.70×10^-11^ | *HEG1, MUC13, OSBPL11, SLC12A8, ITGB5, KALRN* |

LTN, left teat number at birth; RTN, right teat number at birth; SNPs, single nucleotide polymorphisms; chr, chromosome; Pos, Position.

**Table S2** Significant InDels of born teat number traits.

| Traits | InDels | Pos (bp) | *P*-value | Candidate Gene |
| --- | --- | --- | --- | --- |
| LTN | chr1:92929458 | 92929458 | 9.46×10^-13^ | *MTO1, MB21D1* |
|  | chr1:143242249 | 143242249 | 3.70×10^-8^ | *OTUD7A, KLF13, TRPM1* |
|  | chr2: 64358815 | 64358815 | 2.84×10^-13^ | *PTGER1, PKN1, ADGRE5, TECR* |
|  | chr2:64521647 | 64521647 | 3.10×10^-7^ | *TECR, ADGRL1* |
|  | chr3:30147185 | 30147185 | 1.12×10^-7^ | *SHISA9, SNX29, CPPED1* |
|  | chr:94281324 | 94281324 | 8.01×10^-10^ | *PRKCE* |
|  | chr3:132242431 | 132242431 | 7.02×10^-10^ | *ALKAL2, ACP1, SH3YL1, MYT1L* |
|  | chr6:56469155 | 56469155 | 4.79×10^-12^ | *TMC4, PRKCG* |
|  | chr7:79259869 | 79259869 | 3.52×10^-7^ | —— |
|  | chr8: 41462161 | 41462161 | 9.78×10^-9^ | *PDGFRA, KIT, KDR* |
|  | chr10:15308901 | 15308901 | 9.48×10^-17^ | *PLD5, CDC42BPA* |
|  | chr10:19469826 | 19469826 | 1.13×10^-9^ | *CAPN2, SMYD3* |
|  | chr12:37228932 | 37228932 | 1.00×10^-7^ | *APPBP2, USP32* |
|  | chr13:10143939 | 10143939 | 8.22×10^-18^ | *UBE2E2* |
|  | chr15:97926 | 97926 | 2.94×10^-7^ | *CACNB4, ARL5A, STAM2, NEB* |
|  | chr17:10383229 | 10383229 | 4.16×10^-11^ | *GOLGA7, GINS4, GPAT4, ZMAT4, ANK1, SFRP1* |
| RTN | chr7:79596224 | 79596224 | 2.34×10^-9^ | —— |
|  | chr12:40415633 | 40415633 | 1.31×10^-7^ | *LIG3, CCT6B, CCL1, SLFN11, RFFL, TMEM132E, AP2B1* |
|  | chr13:135299486 | 135299486 | 2.57×10^-7^ | *HEG1, MUC13, UMPS, OSBPL11, SNX4* |
|  | chr15:94681349 | 94681349 | 1.41×10^-7^ | *INPP1, HIBCH, MFSD6, PMS1, ORMDL1* |
|  | chr17:7548872 | 7548872 | 1.04×10^-8^ | *TRIML1, TRIML2, ZFP42* |
| TTN | chr7:79596224 | 79596224 | 1.91×10^-9^ | —— |
|  | chr8:674683 | 674683 | 1.07×10^-7^ | *NELFA, NAT8L, GAK, FAM53A, LETM1, POLN* |
|  | chr8: 85382584 | 85382584 | 1.52×10^-8^ | *IL15, INPP4B* |
|  | chr14:55026440 | 55026440 | 6.30×10^-9^ | *ERO1B, GPR137B, NID1, LYST* |
|  | chr15:94681349 | 94681349 | 1.24×10^-8^ | *ORMDL1, NEMP2* |

**Table S3** Significant SVs of born teat number traits

| Traits | Chr | Start | End | Type | *P*-value | Candidate Gene |
| --- | --- | --- | --- | --- | --- | --- |
| LTN | 1 | 17841781 | 17841835 | DEL | 3.69×10^-9^ | *SAMD5, SASH1* |
|  | 1 | 103223565 | 103223636 | DEL | 6.15×10^-9^ | *DCC, MBD2* |
|  | 3 | 27195761 | 27195811 | DEL | 8.13×10^-7^ | *SMG1, XYLT1* |
|  | 4 | 5698461 | 5698518 | DEL | 1.68×10^-8^ | —— |
|  | 6 | 101868877 | 101869176 | DEL | 8.26×10^-7^ | *AKA1N1, DLGAP1, EPB41L3* |
|  | 10 | 36310727 | 36310792 | DEL | 4.31×10^-9^ | *LINGO2* |
|  | 11 | 43078271 | 43078355 | DEL | 3.52×10^-6^ | —— |
|  | 13 | 11234434 | 11234737 | DEL | 4.65×10^-9^ | *NR1D2, THRB* |
|  | 14 | 23273377 | 23274212 | DEL | 3.05×10^-7^ | *PGAM5, POLE, EP400, MMP17, PXMP2* |
|  | 14 | 70909432 | 70909706 | DEL | 4.19×10^-9^ | *CTNNA3, MYPN* |
|  | 15 | 46337339 | 46337399 | DEL | 1.25×10^-8^ | *SLC25A4, SNX25, SORBS2, UFSP2* |
|  | 17 | 30348461 | 30348769 | DEL | 4.69×10^-9^ | *GZF1, NAPB, ACSS1, SYNDIG1* |
| RTN | 10 | 36310727 | 36310792 | DEL | 1.39×10^-10^ | *LINGO2* |
|  | 13 | 131777194 | 131777470 | DEL | 6.52×10^-7^ | *ACAP2, ATP13A3* |
| TTN | 2 | 8384754 | 8385036 | DEL | 1.14×10^-10^ | *SLC22A8, STIP1, MARK2* |
|  | 3 | 27195761 | 27195811 | DEL | 1.52×10^-10^ | *\APBB1, ARFIP2, ARL6IP1, RPS15A, SMG1, XYLT1* |
|  | 9 | 3070619 | 3070937 | DEL | 1.23×10^-6^ | *SMPD1, DCHS1, TAF10, TRIM3* |
|  | 10 | 36310727 | 36310792 | DEL | 1.93×10^-11^ | *LINGO2* |
|  | 11 | 39671912 | 39672014 | DEL | 3.92×10^-6^ | *PCDH9* |
|  | 13 | 179402044 | 179402362 | DEL | 3.15×10^-6^ | *RBM11, NRIP1, ROBO2* |

**Table S4** Significant SNPs of functional teat number traits.

| Traits | SNPs | Pos (bp) | *P*-value | Candidate Gene |
| --- | --- | --- | --- | --- |
| FLTN | chr13: 13706701 | 13706701 | 1.29×10-^10^ | *LRRC3B, NEK10, SLC4A7* |
| FRTN | chr13: 135700692 | 135700692 | 3.47×10-^10^ | *HEG1, MUC13, UMPS, SLC12A8, ITGB5, KALRN* |

**Table S5** Significant InDels of functional right teat number traits.

| Traits | InDels | Pos (bp) | *P*-value | Candidate Gene |
| --- | --- | --- | --- | --- |
| FRTN | chr4:109078512 | 109078512 | 5.80×10-8 | *WDR77*, *PIFO*, *KCNA3*, *DDX20*, *RAP1A*, *TMIGD3*, *ADORA3*, *ATP5F1*, *OVGP1*, *CHIA*, *CHI3L2*, *DENND2D*, *DRAM2*, *LRIF1*, *KCNA2*, *KCND3*, *FAM212B*, *CEPT1*, *CD53* |
|  | chr7:99210660 | 99210660 | 4.06×10^-8^ | *FLVCR2, TGFB3, TTLL5, IFT43, ESRRB* |
|  | chr13:132019431 | 132019431 | 6.33×10^-8^ | *FAM43A, LSG1, PPP1R2, APOD, TMEM44, ACAP2, XXYLT1* |

**Table S6** Significant KEGG pathways and GO terms associated with teat number traits in pigs.

| Traits | Term | Database | ID | Candidate Gene |
| --- | --- | --- | --- | --- |
| Born teat number | Wnt signaling pathway | Gene Ontology | GO:0016055 | *MKS1, RNF43, YAP1, SULF1* |
|  | animal organ formation | Gene Ontology | GO:0048645 | *MKS1, LRP2, SULF1* |
|  | epithelium development | Gene Ontology | GO:0060429 | *VEZF1, MKS1, HS3ST3B1, HEG1, LRP2, YAP1, SULF1, PRKDC* |
|  | hormone-mediated signaling pathway | Gene Ontology | GO:0009755 | *THRB, ACSL1, UFSP2* |
|  | cGMP-PKG signaling pathway | KEGG PATHWAY | KEGG:04022 | *ADORA3, ITPR2, TRPC6* |
|  | JAK-STAT signaling pathway | KEGG PATHWAY | KEGG:04630 | *IL15, PDGFRA, SOCS5* |
|  | PI3K-Akt signaling pathway | KEGG PATHWAY | KEGG:04151 | *ITGB5, PDGFRA, PKN1* |
| Functional teat number | cellular process involved in reproduction in multicellular organism | Gene Ontology | GO:0022412 | *DDX20, WDR77* |
|  | regulation of developmental process | Gene Ontology | GO:0050793 | *CD53, RAP1A, TGFB3, WDR77* |
|  | gland development | Gene Ontology | GO:0048732 | *TGFB3, WDR77* |
|  | ECM-receptor interaction | KEGG PATHWAY | KEGG:04512 | *ITGB5* |
|  | Biosynthesis of cofactors | KEGG PATHWAY | KEGG:01240 | *UMPS* |
